# Right vertical axillary incision vs. median sternotomy for congenital ventricular septal defect repair in children: a propensity score-matched study

**DOI:** 10.3389/fcvm.2024.1527042

**Published:** 2025-01-07

**Authors:** Lijuan Liu, Chenhan Wang, Jie Dong, Jiayi Lin, Mingxiao Liu, Wei Li, Debin Zeng, Xiaohui Yang, Xicheng Deng

**Affiliations:** ^1^ Department of Cardiothoracic Surgery, The Affiliated Children’s Hospital of Xiangya School of Medicine, Central South University (Hunan Children’s Hospital), Changsha, Hunan, China; ^2^ Pediatrics Research Institute of Hunan Province, The Affiliated Children’s Hospital of Xiangya School of Medicine, Central South University (Hunan Children’s Hospital), Changsha, Hunan, China; ^3^ Department of Pediatrics, Affiliated Hospital of Xiangnan University, Chenzhou, Hunan, China

**Keywords:** right vertical axillary incision, median sternotomy, congenital heart disease, congenital ventricular septal defect, children

## Abstract

**Objective:**

To retrospectively assess the outcomes of open-heart repair for ventricular septal defect in children using a right vertical axillary incision compared to median sternotomy.

**Method:**

From January 2022 to May 2023, children who underwent open-heart surgery for the repair of congenital ventricular septal defect in our department were selected for a propensity score-matched study. The propensity score matching method was utilized to pair children in the right vertical axillary incision group with those undergoing surgery via median sternotomy at a 1:1 ratio, based on age and weight.

**Results:**

There were 35 cases in each group. In the right vertical axillary incision group, the median age was 15 (7–40) months and the median weight was 8 (7–12) kg. In the median sternotomy group, the median age was 7 (3–37) months and the median weight was 7 (5–14) kg. The age (*Z* = −1.871, *p* = 0.061) and weight (*Z* = −1.462, *p* = 0.144) of the two groups showed no significant differences. The right vertical axillary incision group exhibited a significantly shorter incision length compared to the median sternotomy group (*p* *<* 0.001). Additionally, the median postoperative drainage was lower in the right vertical axillary incision group than in the median sternotomy group (*p* *=* 0.044), indicating statistical significance. No significant differences were observed between the groups concerning operation time (*p* *=* 0.565), bypass time (*p* *=* 0.855), cross-clamp time (*p* *=* 0.204), oxygenation index one hour post-surgery (*p* *=* 0.651), pleural effusion at 12 h post-surgery (*p* *=* 0.470), abnormal postoperative electrocardiogram (*p* *=* 0.452), cardiac intensive care unit duration (*p* *=* 0.211), or length of hospital stay (*p* *=* 0.095). The hospitalized children were followed up for 3 months to 1 year, during which there were no fatalities or complications.

**Conclusion:**

Open-heart repair of congenital ventricular septal defect through a right vertical axillary incision is a safe and effective surgical technique that minimizes surgical trauma and enhances aesthetic outcomes.

## 1 Introduction

Congenital ventricular septal defect (VSD) is the most prevalent form of congenital heart disease (CHD), accounting for more than 20% of all congenital heart diseases (1). The traditional surgical approach involves median sternotomy, which has been shown to yield satisfactory results. Nonetheless, the scars resulting from this procedure can have substantial psychological and social implications, especially for children and adolescents (2). With advancements in cardiac surgery, anesthesia, and cardiopulmonary bypass techniques, there is an increasing interest in minimizing perioperative trauma and postoperative complications, while simultaneously enhancing the aesthetic quality of surgical incisions. This trend has prompted the investigation of alternative surgical incisions, including upper sternotomy, right anterior thoracotomy, right posterior thoracotomy, and axillary incisions, which may be either horizontal or vertical (3–8). The present study investigates the safety and cosmetic outcomes of open-heart surgery via a right vertical axillary incision in pediatric patients with ventricular septal defects. This research aims to contribute to the advancing field of minimally invasive cardiac surgery, which seeks to balance therapeutic efficacy with aesthetic results.

## 2 Materials and methods

After ethics approval was granted from the hospital's ethics committee (No.HCHLL-2024-309), this study presented a retrospective propensity score-matched analysis of children who underwent open-heart surgery for congenital VSD in our department from January 2022 to May 2023. Written informed consent was obtained from all parents or legal guardians for minors. Children who received surgery via vertical right axillary thoracotomy were classified as the right vertical axillary incision group, whereas those who underwent median sternotomy were designated as the median sternotomy group. To ensure equilibrium in prognostic factors between the two groups, age and weight were employed as matching variables, adhering to the propensity score method with a caliper width of 0.02 and utilizing the nearest available matching technique. Cases from the right vertical axillary incision group were matched 1:1 with corresponding cases from the median sternotomy group.

There are various classification methods for congenital VSD, and no unified standard currently exists. We adopted the widely recognized Anderson classification system, which categorizes congenital VSD into perimembranous, muscular, and doubly committed and juxta-arterial types (9). Based on preoperative transthoracic echocardiography and transesophageal echocardiography (TEE) results, cases with complex congenital heart defects, such as coarctation of the aorta, transposition of the great arteries, pulmonary artery stenosis or atresia, double outlet right ventricle, or severe valvular diseases, were excluded. Preoperative evaluations, including infection markers, liver and kidney function tests, and coagulation function tests, confirmed that all subjects were within normal ranges, with no contraindications for surgery. Surgical procedures for both groups were conducted by the same team of surgeons, with TEE utilized to monitor the repair of defects. Postoperative care in the cardiac intensive care unit (CICU) was administered by the same medical team. The safety and efficacy of the right vertical axillary incision were evaluated by comparing operative details, postoperative recovery, and complication rates between two groups. The fundamental clinical characteristics of the pediatric cohorts are presented in Table 1.

**Table 1:** Clinical information of the study participants.

	Median sternotomy group	Right vertical axillary incision group	*Z*/*χ*^2^	*p*
	*n* = 35	*n* = 35		
Gender (male/female)	16/19	16/19	<0.001	0.99
Weight (kg)	7 [5, 14]	8 [7, 12]	−1.462	0.144
Age (months)	7 [3, 37]	15 [7, 40]	−1.871	0.061
VSD diameter (mm)	10 [8, 12]	10 [8, 10]	−0.157	0.876
VSD type			6.437	0.011
Perimembranous	25 (71.4)	33 (94.3)		
Doubly committed and juxta-arterial	10 (28.6)	2 (5.7)		
PH(Yes/No)	11/24	10/25	0.068	0.794
Preoperative mechanical ventilation (Yes/No)	2/33	1/34	0.348	0.555
Preoperative electrocardiogram			1.014	0.313
Normal	35 (100)	34 (97.1)		
Abnormal	0 (0)	1 (2.9)		
Preoperative NT-proBNP (pg/ml)	705.5 [152.75, 1,841]	369 [180.7, 965.4]	−1.068	0.285
Preoperative Hb (g/L)	111 [102, 120]	117 [110, 124]	−1.992	0.046

Hb, hemoglobin; NT-proBNP, N-terminal pro-brain natriuretic peptide; PH, pulmonary hypertension; VSD, ventricular septal defect.

### 2.1 Surgical techniques

**Figure 1:**
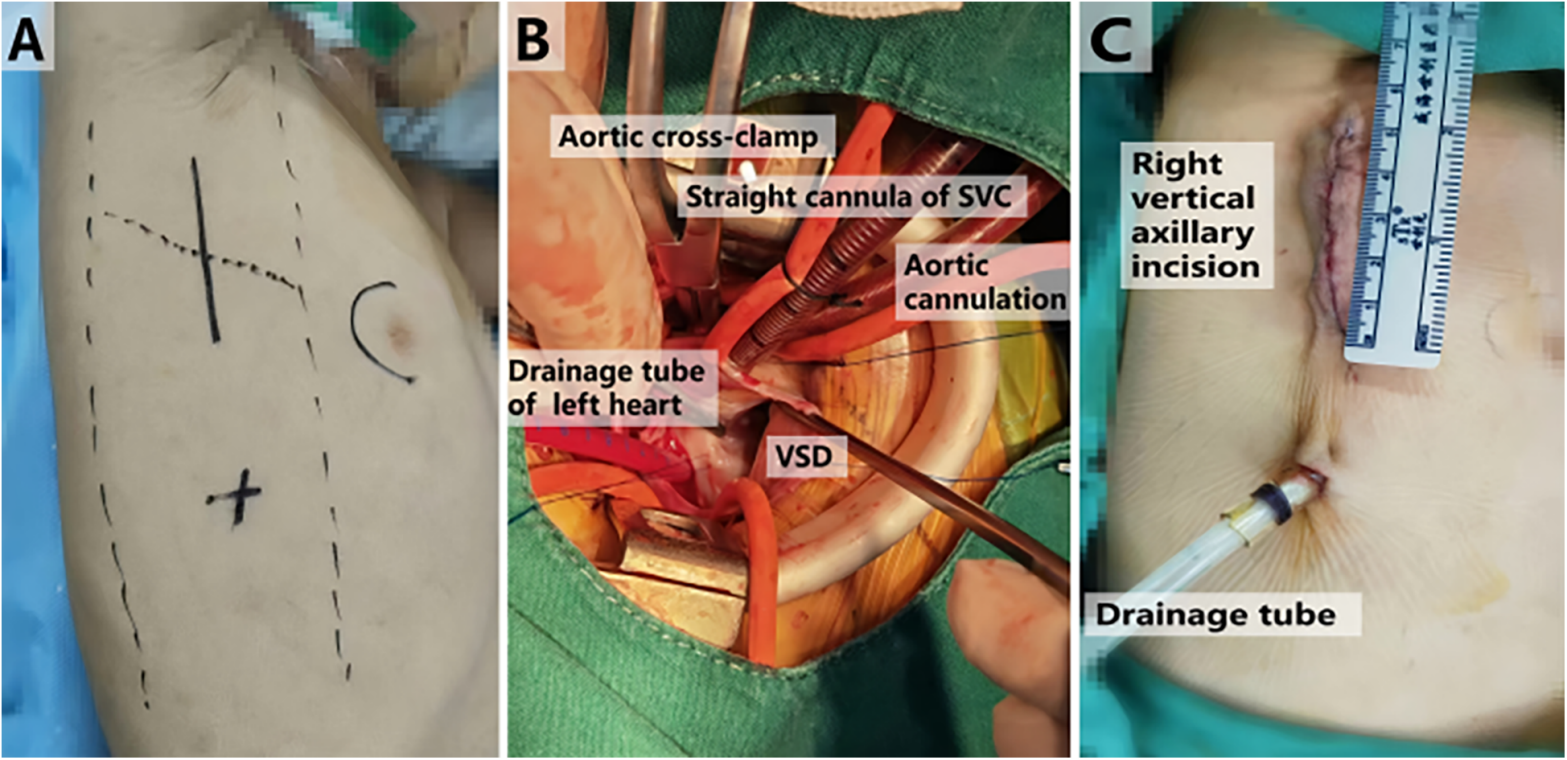
Right vertical axillary incision at different times in surgery. **(A)** Perform localization before surgery. **(B)** Repair VSD during surgery. **(C)** Measure the incision length after surgery. VSD, ventricular septal defect; SVC, superior vena cava.

Right Vertical Axillary Incision Group: Following endotracheal intubation under general anesthesia and TEE assessment, the patient is placed in the left lateral decubitus position. The right hemithorax is elevated to 70 degrees, with the right arm secured above the head, either fixed or suspended from a head frame. A longitudinal incision is initiated at the midpoint of the lower edge of the right axilla, extending to the intersection of the fifth and sixth ribs (Figure 1A). The anterior serratus muscle is dissected along its fibers, and entry into the chest cavity is achieved through either the fourth or fifth intercostal space after positioning a right bronchial blocker by the anesthesiologist. An incision protector and spreader are employed to widen the surgical opening. A longitudinal incision in the pericardium is made approximately 1–2 cm anterior to the phrenic nerve, and silk sutures are used to elevate the right pericardium and the pericardium surrounding the ascending aorta, thereby providing full exposure of the heart. After heparinization, routine aortic cannulation is performed through the same incision. One straight cannula is inserted into the superior vena cava via the right atrial appendage, while another straight cannula is inserted into the inferior vena cava, establishing extracorporeal circulation. Once the activated clotting time of whole blood exceeds 480 s, extracorporeal circulation is initiated. The superior and inferior vena cava, as well as the ascending aorta, are occluded, and del Nido cardioplegia solution is infused at the root of the aorta to induce cardiac arrest. For perimembranous VSD, an incision is made in the right atrium, followed by the application of a 6-0 polypropylene suture to secure a pericardial patch for repair (Figure 1B). For doubly committed and juxta-arterial VSD, a transverse incision in the pulmonary artery is performed, utilizing the same suture and patch material for repair. Upon completion of the procedure, the surgical field is de-aired, and the aortic cross-clamp is released to reestablish normal blood flow. Once the heart resumes a regular rhythm, the extracorporeal circulation system is evacuated, and the pericardium is partially closed with sutures. If bradycardia, atrioventricular block, or other issues occur after cardiac resumption, a temporary pacing wires should be implanted for pacing. Postoperatively, the adequacy of the correction is reassessed using TEE, followed by closure of the surgical incision. A thoracic drainage tube is positioned at the upper border of the sixth rib, just below the incision site (Figure 1C), and postoperative care is administered in the CICU.

Median sternotomy group: The patient is positioned supine and undergoes general anesthesia with endotracheal intubation. After TEE assessment, a measurement is taken along the sternal midline extending from the thoracic angle to the xiphoid process. A longitudinal incision is made to divide the sternum, and the cut edges are treated with bone wax to minimize bleeding. A sternal retractor is then employed to separate the sternum. The pericardium is incised in a T-shape, suspended, and affixed to the chest wall, allowing for full exposure of the heart. A 6-0 polypropylene suture and an autologous pericardial patch are utilized to repair the defect. The surgical approach for various types of congenital VSD conforms to the protocol established for the right vertical axillary incision group.

### 2.2 Observation indicators

The operative incision length, operation time, bypass duration, cross-clamp duration, postoperative mechanical ventilation duration, oxygenation index post-surgery, incidence of pleural effusion, early postoperative drainage, duration of the drainage tube retention, postoperative analgesia, levels of postoperative hemoglobin (Hb), white blood cells (WBC), C-reactive protein (CRP), N-terminal pro-brain natriuretic peptide (NT-proBNP), time spent in the CICU, and overall length of hospital stay were compared between the two groups.

### 2.3 Statistical analysis

Data normality was assessed using the Shapiro-Wilk test, with a *p* > 0.05 suggesting a normal distribution. For normally distributed data, results were expressed as means ± standard deviations and analyzed using the *t*-test. Conversely, when the *p* ≤ 0.05, indicating a non-normal distribution, data were presented as medians with interquartile ranges and compared using nonparametric tests. Categorical data were analyzed using the Chi-square test. All statistical analyses were conducted using SPSS version 19.0 software, with a *p* < 0.05 was considered statistically significant.

## 3 Results

A total of 35 cases were included in the right vertical axillary incision group, consisting of 16 males and 19 females, with a median age of 15 months (7–40 months) and a median weight of 8 kg (7–12 kg). Employing the propensity score method with a 1:1 matching ratio, 35 cases from a pool of 241 children who underwent surgery via median sternotomy were selected for the median sternotomy group. This cohort also comprised 16 males and 19 females, with a median age of 7 months (3–37 months) and a median weight of 7 kg (5–14 kg). Post-matching analysis revealed no significant differences in gender, age, weight, and other demographic factors between the two groups (*p* > 0.05).

No statistically significant differences were observed in the congenital VSD diameter, preoperative NT-proBNP levels, electrocardiogram results, and the presence of concomitant pulmonary hypertension (PH) (*p* > 0.05). Perimembranous VSD types were predominant in the right vertical axillary incision group, with only 2 cases of doubly committed and juxta-arterial VSD, indicating a statistically significant difference (*p* *=* 0.011). Furthermore, preoperative Hb levels were significantly higher in the right vertical axillary incision group compared to the median sternotomy group (*p* *=* 0.046) (Table 1).

The surgical outcomes for the two groups were as follows: the median operation duration for the right vertical axillary incision group was 150 min (135–169 min), with a median bypass duration of 64 min (56–72 min) and a median cross-clamp duration of 35 min (29–41 min). In the median sternotomy group, the median operation duration was 145 min (135–165 min), with a median bypass duration of 65 min (55–74 min) and a median cross-clamp duration of 33 min (25–38 min). The operative parameters indicated no significant differences between the groups (*p* > 0.05), as detailed in Table 2. Both groups successfully underwent surgery without any complications or residual shunt. There were no recorded instances of deaths or major complications during the early postoperative period.

**Table 2:** Operative parameters of the study participants.

	Median sternotomy group	Right vertical axillary incision group	*Z*/χ^2^	*p*
	*n* = 35	*n* = 35		
Operation duration (minutes)	145 [135, 165]	150 [135, 169]	−0.576	0.565
Bypass duration (minutes)	65 [55, 74]	64 [56, 72]	−0.182	0.855
Cross-clamp duration (minutes)	33 [25, 38]	35 [29, 41]	−1.27	0.204

Postoperative conditions were compared between the two groups, revealing no statistically significant differences in the oxygenation index at 1 h (*p* *=* 0.651), 12 h (*p* *=* 0.805), and 24 h (*p* *=* 0.404) postoperatively. Additionally, there were no significant differences in the incidence of pleural effusion (*p* *=* 0.470), WBC count (*p* *=* 0.306), CRP levels (*p* *=* 0.874), NT-proBNP levels (*p* *=* 0.927) at 12 h postoperatively, and electrocardiographic abnormalities (*p* *=* 0.452). Furthermore, no statistically significant differences were observed regarding mechanical ventilation duration (*p* *=* 0.235), drainage tube retention duration (*p* *=* 0.545), CICU duration (*p* *=* 0.211), length of hospital stay (*p* *=* 0.095), and hospitalization expenses (*p* *=* 0.125).

The operative incision length in the right vertical axillary incision group was 6 (5, 6) cm, which was significantly shorter than that of the median sternotomy group at 5 (4, 5) cm, with a statistically significant difference (*p* < 0.001). The Hb level in the right vertical axillary incision group one hour post-operation was 122 (117, 126) g/L, higher than that of the median sternotomy group at 113 (104, 123) g/L, and this difference was also statistically significant (*p* = 0.005). The postoperative drainage at 12 h following surgery for the right vertical axillary incision group was 55 ml (45–70 ml), which was significantly lower than that observed in the median sternotomy group (70 ml; 50–90 ml) (*p* *=* 0.044), as presented in Table 3.

**Table 3:** Postoperative information of the study participants.

	Median sternotomy group	Right vertical axillary incision group	*Z*/χ^2^	*p*
	*n* = 35	n = 35		
Operative incision length (cm)	6 [5, 6]	5 [4, 5]	−5.836	<0.001
Oxygenation index				
1 h after surgery	300 [223, 407]	280 [205, 384]	−0.452	0.651
12 h after surgery	517 [284, 559]	452 [352, 564]	−0.247	0.805
24 h after surgery	405.5 [292.25, 563]	448 [371.5, 519]	−0.834	0.404
WBC(10^9^/L)	12 [11, 14]	12 [8, 14]	−1.023	0.306
Hb (g/L)	113 [104, 123]	122 [117, 126]	−2.827	0.005
CRP (mg/ml)	25 [10, 38]	25 [12, 36]	−0.159	0.874
NT-proBNP (pg/ml)	1,940 [1,132.25, 3,542]	1,938 [1,265.5, 2,794]	−0.091	0.927
Electrocardiogram			0.565	0.452
Normal	30 (85.7)	32 (91.4)		
Abnormal	5 (14.3)	3 (8.6)		
Pleural effusion (Yes/No)	18/17	21/14	0.521	0.470
Postoperative drainage (ml)	70 [50, 90]	55 [45, 70]	−2.012	0.044
Drainage tube retention duration (h)	88 [70, 116]	89 [68, 96]	−0.606	0.545
Mechanical ventilation duration (h)	4 [2, 19]	3 [2, 6]	−1.188	0.235
Postoperative Analgesia (Yes/No)	33/2	29/6	2.258	0.133
CICU duration (h)	93 [41, 142]	66 [45, 94]	−1.252	0.211
Length of hospital stay (d)	14 [10, 21]	11 [10, 14]	−1.669	0.095
Hospitalization expenses (yuan)	66,971 [54,759, 76,455]	59,675 [55,116, 65,233]	−1.533	0.125

CICU, cardiac intensive care unit; CRP, C-reactive protein; Hb, hemoglobin; NT-proBNP, N-terminal pro-brain natriuretic peptide; WBC, white blood cells.

The hospitalized children were followed up for 3 months to 1 year, during which there were no fatalities or major complications.

## 4 Discussion

The treatment options for congenital VSD typically depend on the size and location of the defect, the patient's clinical symptoms, and the presence of other cardiac anomalies. Currently, new surgical approaches such as interventional closure, thoracoscopic-assisted surgery, and robot-assisted surgery have been widely adopted (10). For asymptomatic or mildly symptomatic small congenital VSD, observation and regular follow-up are often employed, as most small congenital VSD may close spontaneously with age, especially in muscular VSD. For large congenital VSD, surgical repair remains the first-line treatment (11). Early surgery often yields favorable outcomes, particularly when performed during infancy or early childhood, effectively preventing complications such as pulmonary hypertension and heart failure (12).With the continuous advancements in the diagnosis and treatment of valvular heart disease, the success rate of surgical interventions for simpler forms of valvular defects, such as congenital VSD, has steadily increased. Most patients can achieve near-normal quality of life, while complications and mortality rates have significantly decreased (13, 14). However, the prognosis of surgical treatment is still influenced by multiple factors, including the size and type of the defect, the timing of the surgery, and postoperative complications. Improvements in surgical techniques and perioperative management strategies have greatly facilitated postoperative recovery for patients. However, the psychological impact of visible surgical scars on pediatric patients should not be underestimated (15). Patients and their families demonstrate a strong preference for surgical approaches that prioritize the aesthetic appearance of surgical wounds. As a result, minimizing surgical trauma has become increasingly important in the pursuit of patient-centered care and satisfaction.

Our center has standardized the open-heart repair of atrial septal defects using a small incision under the right axilla, achieving favorable outcomes (16). In this study, we employed a similar approach to address ventricular septal defects via a small incision made under the right axilla. The group undergoing the right vertical axillary incision demonstrated satisfactory postoperative success rates and long-term outcomes comparable to those of the median sternotomy group. Notably, the right vertical axillary incision group exhibited favorable cosmetic results, characterized by the following: (1) The incision length was smaller than that of the median sternotomy group. In this study, the surgical incision length in the right vertical axillary incision group was 6 (5, 6) cm, significantly less than the median sternotomy group, which was 5 (4, 5) cm. The surgical incision can be concealed in the axilla, thus avoiding scars in the center of the sternum, which contributes to improved postoperative aesthetics. Additionally, advances in surgical techniques may further reduce the size of the incision. Reports suggest that for simple defect repairs, an incision measuring 2–4 cm in length is often sufficient (17); (2) The incision is positioned away from the breast tissue and pectoral muscles, thereby not affecting normal breast development and aiding in the prevention of potential deformities, such as an asymmetric chest wall (18, 19); (3) The postoperative wound pain is mild, which benefits the patient's rapid recovery after surgery.

The baseline characteristics of the two study cohorts were largely similar, with participants in both groups choosing to undergo surgery under stable preoperative conditions, without experiencing severe complications and presenting normal preoperative infection markers. A crucial distinction between the groups, however, lies in the type of congenital VSD they exhibited, as the right vertical axillary incision group primarily included patients with perimembranous VSD. The right vertical axillary approach offers limited access to doubly committed and juxta-arterial VSD, which can introduce specific surgical challenges and increase procedural complexity. Consequently, the traditional median sternotomy incision is often preferred for cases involving doubly committed and juxta-arterial VSD. Nevertheless, recent literature indicates that for patients with doubly committed and juxta-arterial VSD, both cardiopulmonary bypass duration and operative duration are significantly reduced when utilizing the right vertical axillary incision (17, 19). As this study marks our first exploration of the right vertical axillary incision in congenital VSD repair, it is conceivable that the application of this technique may be expanded to include other types of congenital VSD, such as doubly committed and juxta-arterial VSD, as our proficiency with this method increases.

This study compared the overall operation duration, bypass duration and cross-clamp duration between the right vertical axillary incision group and the median sternotomy group, finding no statistically significant differences. The right vertical axillary incision group did not undergo intraoperative sternotomy, suggesting that neither surgical approach significantly impacted the overall operation duration. However, the operation duration was slightly longer in the right vertical axillary incision group, which is attributed to the learning curve associated with this technique. Nevertheless, once this method is proficiently mastered, it is anticipated that the operation duration may be comparable to, or potentially even shorter than, that of the median sternotomy approach. This expectation is based on the avoidance of sternotomy and its associated opening and closure in the right vertical axillary incision group.

This study compared chest x-ray results obtained 12 h postoperatively and found a slightly higher incidence of pleural effusion in the right lung than in the median sternotomy group; however, this difference was not statistically significant. Additionally, no significant statistical differences were observed in the postoperative oxygenation index, mechanical ventilation duration, CICU duration, length of hospital stay, or inflammatory indicators between the two groups. These findings indicate that lung conditions do not substantially affect overall treatment outcomes and prognosis.

During the right vertical axillary approach, it is essential to protect the right lung and minimize the adverse effects of mechanical injuries, such as compression. If feasible, techniques such as bronchial occlusion and single-lung ventilation should be employed to their fullest extent. Furthermore, preoperative Hb values indicated that the median sternotomy group had higher levels compared to the right vertical axillary incision group. One hour postoperatively, when patients returned to the CICU, the Hb values in the right vertical axillary incision group were higher than those in the median sternotomy group. Evaluating the postoperative drainage of both groups suggests that the median sternotomy group experienced greater incision exudate and blood loss, likely due to increased blood infiltration resulting from sternal splitting. The thoracotomy approach, by avoiding sternotomy, can prevent damage to bony tissue and effectively reduce surgical trauma.

In comparison to traditional median sternotomy, surgical intervention through a small vertical incision in the right axilla has been associated with a reduced incidence of complications. This approach effectively addresses all heart defects without resulting in residual deformities, thereby validating the safety and efficacy of the technique. The significant reduction in incision length contributes to better scar concealment. Furthermore, the lateral incision spares the bony structures of the chest, which mitigates surgical trauma, alleviates postoperative wound pain to some extent, and fosters the long-term mental well-being of the child (18).

It is noteworthy that the total cost associated with the lateral resection group includes expenses related to single-lung ventilation management during anesthesia, which exceeds those associated with standard double-lung ventilation. Although no statistically significant difference in overall expenses was observed, the group with a right vertical axillary incision demonstrated a tendency towards lower costs. Increasing the sample size may provide a more definitive conclusion.

In the initial stages of performing right vertical axillary incision surgery, the surgical team, including surgeons, anesthesiologists, and cardiopulmonary bypass perfusionists, faces high demands. Surgeons must exercise caution during pericardial incision, taking care to protect the phrenic nerve to avoid diaphragmatic injury that could lead to diaphragmatic paralysis and elevation, thereby preventing respiratory complications. When cannulating the ascending aorta, the deeper location compared to a median sternotomy incision necessitates the use of forceps to grasp the cannula tip, facilitating a smoother cannulation process. During cardiopulmonary bypass, special attention should be given to drainage flow; insufficient drainage requires timely application of vacuum-assist venous drainage. In addition, for anesthesiologists, protecting lung function is particularly important, including low tidal volume mechanical ventilation and preventing mechanical lung injury, to reduce the risks of atelectasis and pneumothorax.

We used a right vertical axillary incision for selected atrial septal defects and gradually accumulated extensive experience during the process (17). This experience laid a solid foundation for performing congenital VSD repairs through a similar incision. Despite gaining considerable procedural experience, surgeons must remain highly cautious and carefully select appropriate patients. Ling Yan et al. pointed out that the right vertical axillary incision is more suitable for children weighing less than 30 kg, thereby improving surgical safety (5). Moreover, research has shown that this surgical approach demonstrates ideal efficacy in congenital VSD repairs for children aged 2–5 years (20). In this study, we found that the maximum weight of children in the right axillary incision group was 20 kg, with a median age of 15 months (ranging from 7–40 months), which is consistent with the clinical experience from other cardiac centers.

Our research has certain inherent limitations. As a single-center study, it only included clinical data of patients treated at our department, and the sample size is relatively small. The right vertical axillary incision group comprised only 35 patients, which may lead to an imbalance in intergroup matching. To minimize selection bias and the influence of multiple factors on the results, we conducted a matching analysis, selecting 35 cases from 241 children who underwent surgery via median sternotomy to form the median sternotomy group. Furthermore, the follow-up period for both groups of patients after discharge ranged from 3 months to 1 year, which is relatively short; therefore, no comparisons were made regarding long-term postoperative complications. Consequently, it is necessary to conduct longer follow-ups to further validate the clinical efficacy of the right vertical axillary incision.

The right vertical axillary incision offers a viable alternative to the median sternotomy approach. However, the right vertical axillary incision is more technically demanding and requires advanced technology. It entails a considerable learning curve and the accumulation of clinical experience. Initially, we recommend this incision technique for selected cases of atrial septal defect and perimembranous ventricular septal defect patients. Even after gaining experience, surgeons should remain cautious when utilizing this incision for more complex cardiac defects, ensuring the careful selection of appropriate candidates for such procedures.

## 5 Conclusion

The right vertical axillary incision represents a safe and effective method for correcting simple congenital cardiac anomalies. This approach significantly reduces surgical trauma and produces enhanced cosmetic outcomes. It serves as a promising alternative to the traditional median sternotomy incision.

## Data Availability

The raw data supporting the conclusions of this article will be made available by the authors, without undue reservation.

## References

[1] Xunmin C Shisen J Jianbin G Haidong W Lijun W. Comparison of results and complications of surgical and Amplatzer device closure of perimembranous ventricular septal defects. Int J Cardiol. (2007) 120(1):28–31. 10.1016/j.ijcard.2006.03.092 17084470

[2] Atalay A Yilmaz M Turkcan BS Ecevit AN Ozler B Azak E Can right infra-axillary vertical thoracotomy make a big difference in surgical technique preference? Heart Lung Circ. (2022) 31(10):1419–24. 10.1016/j.hlc.2022.06.661 35871132

[3] Yoshimura N Yamaguchi M Oshima Y Oka S Ootaki Y Yoshida M. Repair of atrial septal defect through a right posterolateral thoracotomy: a cosmetic approach for female patients. Ann Thorac Surg. (2001) 72(6):2103–5. 10.1016/S0003-4975(01)03086-7 11789801

[4] Silva LdFd Silva Jd Turquetto ALR Franchi SM Cascudo CM Castro RM Horizontal right axillary minithoracotomy: aesthetic and effective option for atrial and ventricular septal defect repair in infants and toddlers. Rev Bras Cir Cardiovasc. (2014) 29(2):123–30. 10.5935/1678-9741.20140028 25140460 PMC4389452

[5] Yan L Zhou ZC Li HP Lin M Wang HT Zhao ZW Right vertical infra-axillary mini-incision for repair of simple congenital heart defects: a matched-pair analysis. Eur J Cardiothorac Surg. (2013) 43(1):136–41. 10.1093/ejcts/ezs280 22619315

[6] Mishaly D Ghosh P Preisman S. Minimally invasive congenital cardiac surgery through right anterior minithoracotomy approach. Ann Thorac Surg. (2008) 85(3):831–5. 10.1016/j.athoracsur.2007.11.068 18291151

[7] Seipelt RG Popov A Danner B Paul T Tirilomis T Schoendube FA Minimally invasive partial inferior sternotomy for congenital heart defects in children. J Cardiovasc Surg (Torino). (2010) 51(6):929–33. 21124291

[8] Yang X Wang D Wu Q. Repair of atrial septal defect through a minimal right vertical infra-axillary thoracotomy in a beating heart. Ann Thorac Surg. (2001) 71(6):2053–4. 10.1016/S0003-4975(01)02470-5 11426806

[9] Anderson RH Baker EJ Redington A Rigby ML Penny D Wernovsky G. Paediatric Cardiology. Philadelphia, PA: Elsevier Health Sciences (2009).

[10] Hong ZN Chen Q Lin ZW Zhang GC Chen LW Zhang QL Surgical repair via submammary thoracotomy, right axillary thoracotomy and median sternotomy for ventricular septal defects. J Cardiothorac Surg. (2018) 13(1):47. 10.1186/s13019-018-0734-5 29783998 PMC5963097

[11] Wang Z Liu W Zhang B. Cardiothoracic Surgery. 1st ed. Beijing: People’s Military Medical Publisher (2003). p. 1851.

[12] Ren L Hao Y Chen X. Effect of right vertical infra-axillary thoracotomy on the repair of ventricular septal defect in children. Chin J Clin Thorac Cardiovasc Surg. (2020) 27(08):870–3. 10.7507/1007-4848.201912057

[13] Schipper M Slieker MG Schoof PH Breur JMPJ. Surgical repair of ventricular septal defect; contemporary results and risk factors for a complicated course. Pediatr Cardiol. (2017) 38(2):264–70. 10.1007/s00246-016-1508-2 27872996 PMC5331080

[14] Scully BB Morales DLS Zafar F McKenzie ED Fraser CD Heinle JS. Current expectations for surgical repair of isolated ventricular septal defects. Ann Thorac Surg. (2010) 89(2):544–9. 10.1016/j.athoracsur.2009.10.057 20103339

[15] Alsarraj MK Nellis JR Vekstein AM Andersen ND Turek JW. Borrowing from adult cardiac surgeons-bringing congenital heart surgery up to speed in the minimally invasive era. Innovations. (2020) 15(2):101–5. 10.1177/1556984520911020 32352905

[16] An K Li S Yan J Wang X Hua Z. Minimal right vertical infra-axillary incision for repair of congenital heart defects. Ann Thorac Surg. (2022) 113(3):896–902. 10.1016/j.athoracsur.2021.01.052 33592183

[17] Yang X Hu Y Dong J Huang P Luo J Yang G Rightvertical axillary incision for atrial septal defect: a propensity score matched study. J Cardiothorac Surg. (2022) 17(1):256. 10.1186/s13019-022-01999-0 36199116 PMC9535985

[18] Prêtre R Kadner A Dave H Dodge-Khatami A Bettex D Berger F. Right axillary incision: a cosmetically superior approach to repair a wide range of congenital cardiac defects. J Thorac Cardiovasc Surg. (2005) 130(2):277–81. 10.1016/j.jtcvs.2005.03.023 16077387

[19] Liu R Rui L Zhang B Lin Y Li S Hua Z. Through tricuspid closure for doubly committed subarterial ventricular septal defect with right vertical subaxillary mini-incision: a matched-pair analysis. Pediatr Cardiol. (2019) 40(6):1247–52. 10.1007/s00246-019-02144-w 31338560

[20] Wang Q Li Q Zhang J Wu Z Zhou Q Wang DJ. Ventricular septal defects closure using a minimal right vertical infraaxillary thoracotomy: seven-year experience in 274 patients. Ann Thorac Surg. (2010) 89(2):552–5. 10.1016/j.athoracsur.2009.11.026 20103340

